# Identification and Characterization of Xlr5c as a Novel Nuclear Localization Protein in Mouse Germ Cells

**DOI:** 10.1371/journal.pone.0130087

**Published:** 2015-06-15

**Authors:** Xin-Jie Zhuang, Wen-hao Tang, Chang-yu Liu, Jin-liang Zhu, Xue Feng, Jie Yan, Ying Lian, Ping Liu, Jie Qiao

**Affiliations:** 1 Center for Reproductive Medicine, Department of Obstetrics and Gynecology, Key Laboratory of Assisted Reproduction, Ministry of Education, Peking University Third Hospital, Beijing 100191, PR China; 2 Department of Urology, the Third Hospital of Peking University, Beijing 100191, PR China; University of Nevada School of Medicine, UNITED STATES

## Abstract

**Background:**

Spermatogenesis is the complex process by which diploid stem cells generate haploid germ cells in gamete production. Members of the Xlr (X-chromosome linked, lymphocyte regulated) superfamily play essential roles in spermatogenesis. The expression, localization and role in spermatogenesis of one such member, Xlr5c, has not been reported previously.

**Methodology/Principal Findings:**

Xlr5c mRNA and protein levels in murine testes and other tissues were investigated using RT-PCR and Western blotting. Xlr5c was abundantly transcribed in mouse testes, particularly during the early stages of spermatogenesis and throughout prophase I in the nuclei of spermatocytes. Xlr5c was specifically localized at synaptonemal complexes(SCs) region in preleptotene and pachytene spermatocytes, as was the homologous Xlr protein Sycp3.

**Conclusions/Significance:**

These results suggest that Xlr5c was abundantly transcribed in germ cells, localized at SCs region, where it may play a potential role during the early stages of spermatogenesis. Identification and characterization of this novel testis protein may offer a new perspective for understanding of the molecular mechanisms involved in germ cell differentiation.

## Introduction

Spermatogenesis is a highly complex process by which diploid cells differentiate into haploid gametes during sexual reproduction. During this process a large number of genes involved in fertility must be highly regulated in both males and females. The targeting of candidate genes with proposed roles in spermatogenesis has provided valuable information. Spermatogenesis is highly organized and involves the expression and interaction of numerous genes [1], but mammalian spermatogenesis is still poorly understood at the molecular level. Identification of genes and loci involved in mammalian spermatogenesis has relied on alternative approaches[2]. The X-chromosome is known to play a crucial role in the development of sexually selected characteristics, and may perform a universal role in male/female fertility that involves regulating the expression of a number of specific genes. Surprisingly, many genes involved in spermatogenesis in mice are X-linked and are expressed exclusively in males.

Members of the Xlr (X-linked, lymphocyte regulated) family share a Cor1 domain that was first identified in terminally differentiated B lymphoid cells [3,4] and functions in spermatogenesis. In all, there are 30 ~ 40 members of the Xlr superfamily including the XLRs themselves, along with synaptonemal complex protein 3 (Sycp3), Sycp3-like X-linked 2 (Slx2), Sycp3-like X-linked (Slx/Xmr), SLX-like1 (Slxl1) and Sycp3-like Y-linked (Sly).

Sycp3 is essential for both formation and maintenance of the lateral elements(LEs) of the synaptonemal complexs (SCs)[5]. Sycp3 includes the conserved Cor1 domain and is required for male fertility. Sycp3 male knockout mouse germ cells die around the zygotene stage of meiosis[6]. Slx2 is highly homologous with Sycp3, is highly expressed in the nuclei of spermatocytes, and may be involved in homologous recombination, double strand repair and sex chromosome inactivation during meiosis[7]. Xlr encodes a 30 kDa nuclear protein expressed in the nucleus of oocytes during prophase of meiosis I and colocalized with the matrix-associated region-binding Satb1 protein[8]. Slx/Xmr was originally described as a testis-specific nuclear protein that is expressed during the male meiotic prophase; however, it has since been shown that Slx encodes a spermatid cytoplasmic protein, and Slx-deficient mice were sterile [9,10,11]. The novel acrosomal protein Slx1 interacts with Dkkl1 and is involved in fertilization in mice [12]. Sly is expressed in spermatids during spermatogenesis and also interacts with Dkkl1 which is involved in fertilization[13]. Spermatozoon differentiation is severely impaired in Sly-deficient mice, and the heads of the spermatozoa are abnormal, which may affect their ability to penetrate the zona pellucida [14]. Xlr3 and Xlr4 comprise a variant subgroup of the Xlr gene family first identified by cDNA cloning [4,15]. Xlr3a and Xlr4b are expressed in the testes and exhibit imprinted expression in the brain[16,17,18,19].

Xlr5c, which includes the Cor1 domain is a member of the Xlr5 subfamily, but the exact functions and molecular mechanisms of this protein are unknown. In this study, Xlr5c was found to be abundantly transcribed in mouse testes, specifically in the nuclei of germ cells. Xlr5c was identified and localized at SCs region, as was previously shown for the homologous Sycp3 during the prometaphase I stage of spermatogenesis. Sycp3 is essential for lateral elements of SCs and spermatogenesis. These results strongly indicate that Xlr5c may involved in spermatogenesis.

## Materials and Methods

### Animals and reagents

Animals were obtained from the Experimental Animal Center, Chinese Academy of Sciences. CHO cells were from the laboratory of Professor Chun-sheng Han, Institute of zoology, Chinese Academy of Sciences. All animal experiments were performed in accordance with the NIH Guide for the Care and Use of Laboratory Animals issued by the Peking University Third Hospital, Beijing. The protocol was approved by the Institutional Animal Care and Use Committee (IACUC) of the Peking University Third Hospital (Protocol Number: 2013SZ021). All mice were bred in a pathogen-free environment with water permanently available, and were fed a mouse breeder diet and maintained at constant temperature and humidity under 12:12 light/dark cycles. Mice were killed by cervical dislocation.

All chemicals and equipment for electrophoresis and transfer were purchased from Bio-Rad Laboratories (Carlsbad, CA) or Sigma (St. Louis, MO) unless specified otherwise. Matchmaker library construction and screening kits were purchased from Invitrogen and Clontech (San Jose, CA). FITC-conjugated and TITC-conjugated anti-rabbit antibodies were supplied by Jackson Laboratories (Cambridge, UK). Primers used for PCR were synthesized by Invitrogen (Table 1).

**Table 1:** Primer pairs used in this study.

Name	Usage	Product Length	Sequence (5'-3')
Xlr5c	RT-PCR	381bp	Sense/ *Bam HI*: ACA GGATCC ATGCAAGAGGCCAGGGAG Antisense/ *XhoI*: ACT CTCGAG GTGGTACACCGACCCAAAT
Xlr5c-GST	Sub-Cloning into pGEX-4T-1	55-127AA	Sense/ *Bam HI*: ACA GGATCC ATGCAAGAGGCCAGGGAG Antisense/ *XhoI*: ACT CTCGAG GTGGTACACCGACCCAAAT
Xlr5c -GFP	Sub-Cloning into pEGFP-N1	1-179AA	Sense/ *XhoI*: ACT CTCGAG ATGCAAGAGGCCAGGGAG Antisense/*BamHI*: ACA GGATCC ccagaccttcaaaaatgT
Gapdh	RT-PCR	439bp	Sense: ACCACAGTCCATGCCATCAC Antisense: TCCACCACCCTGTTGCTGTA

### RNA isolation and RT-PCR

Total RNA from mouse organs including testes, ovary, liver, stomach, small intestine, heart, kidney, lung, uterus and muscle were isolated using TRIzol reagent (Invitrogen) in accordance with the manufacturer’s instructions. For RT-PCR, total RNA was used as a template for reverse transcription using the prime Script 1^st^ strand cDNA synthesis Kit (TAKARA, D6110A) and the Superscript III reverse transcriptase (Invitrogen) in a 15 μl reaction as described previously [7]. Amplification was carried out for 30 cycles, with an annealing temperature of 60°C for the Xlr5c primers. The products of RT-PCR and PCR were analyzed by agarose gel electrophoresis and stained with ethidium bromide. Relative amounts of cDNA were normalized against glyceraldehyde 3-phosphate dehydrogenase (Gapdh).

### Recombinant protein expression and antibody production

The gene encoding Xlr5c (residues 55~127aa) was subcloned into the pGEX-4T1 vector (Pharmacia Biosciences) using primers synthesized by Invitrogen (Table 1). The GST fusion protein was expressed in *E*. *coli* strain BL21 and purified using a GSTrap FF column (Amersham Pharmacia) according to the manufacturer’s instructions. Purified protein (500 μg) was emulsified in complete Freund’s adjuvant and injected into healthy rabbits, and a further three boosting injections of 500 μg were performed at 3-week intervals. Polyclonal antisera were recovered from rabbit blood using standard methods. Antibodies were purified by affinity purification using a protein-G column as previously described [12].

### Immunohistochemistry and immunocytochemistry

Immunohistochemistry was performed as described [20]. Briefly, 8 μm frozen sections of mouse testes were fixed immediately in 4% paraformaldehyde for 15 min at room temperature. After blocking, the sections were incubated with affinity-purified Xlr5c antibody (diluted 1:200 in blocking buffer) or pre-immune rabbit serum (negative control) for 1 hour at room temperature. A FITC-conjugated anti-rabbit secondary antibody (Jackson Laboratories, Bar Harbor, ME) was then used at a dilution of 1:500.

Chromosome spreading was performed on primary spermatocytes using the drying-down technique [7]. Briefly, mice were killed and testes were dissected, and seminiferous tubules were isolated and kept in a hypotonic extraction buffer (30 mM Tris, 50 mM sucrose, 17 mM trisodium citrate dihydrate, 5 mM EDTA, 0.5 mM DTT, 0.5 mM PMSF, pH 8.2) for 30 ~ 60 min. Subsequently, the tubules were torn into small pieces using fine forceps and placed in 100 mM sucrose solution. The cell suspension was mixed with 3.7% paraformaldehyde solution and spread on a clean glass slide. Slides were washed twice for 2 min in 0.4% Photoflo (Kodak), dried at room temperature and stained using anti-γH2AX (1:200, Upstate), anti-Fem1b (1:400) and anti-Xlr5c (1:400). Secondary antibody (FITC- or TRITC-conjugated anti-rabbit (Jackson Laboratories, Bar Harbor, ME) was then applied at a dilution of 1:500.

### Confocal fluorescence and immunohistochemistry

The entire mouse Xlr5c coding sequence was cloned in-frame into the pEGFP-N1 vector, and Xlr5c-GFP-fusion protein was expressed in CHO cells cultured on glass cover slips using Lipofectamine 2000 (Invitrogen) according to the manufacturer’s instructions. Twenty four hours after transfection, cells were fixed with 4% paraformaldehyde for 15 min and permeabilized with 0.2% Triton X-100 for 10 min. After washing with PBS, DAPI (Sigma–Aldrich) was added at a final concentration of 1 μg/ml for 10 min, and samples were mounted on microscope slides. Samples were visualized using a laser confocal microscope (Zeiss). Anti-Xlr5c antibody (1:200) and TRITC-conjugated anti-rabbit secondary antibody (1:200) were used to confirm the fusion protein.

### Western blot analysis

Western blotting was performed using standard protocols. Mouse testes and other tissues were homogenized in RIPA lysis buffer (50 mM Tris, pH 7.4, 10 mM MgCl_2_, 150 mM NaCl, 1%NP-40, 1 mM sodium orthovanadate, 1 mM NaF), and the protein concentration was determined using the Bradford Reagent (BioRad). An equal amount of protein (50 ~ 100 μg total protein/lane) were loaded and separated by SDS–PAGE. Following transfer to nitrocellulose membranes (Amersham Biosciences AB, Uppsala, Sweden), membranes were blocked in TBST (0.5% Tween-20 in TBS) containing 5% nonfat milk powder for 1 h and incubated overnight with a 1:200 dilution of anti-Xlr5c antibody in TBST. After washing three times with TBST, membranes were incubated with HRP-conjugated secondary antibody, and protein expression levels were measured using Image-Pro plus 5.1.

## Results

### Xlr5 is a subfamily of the Xlr family

**Fig 1:**
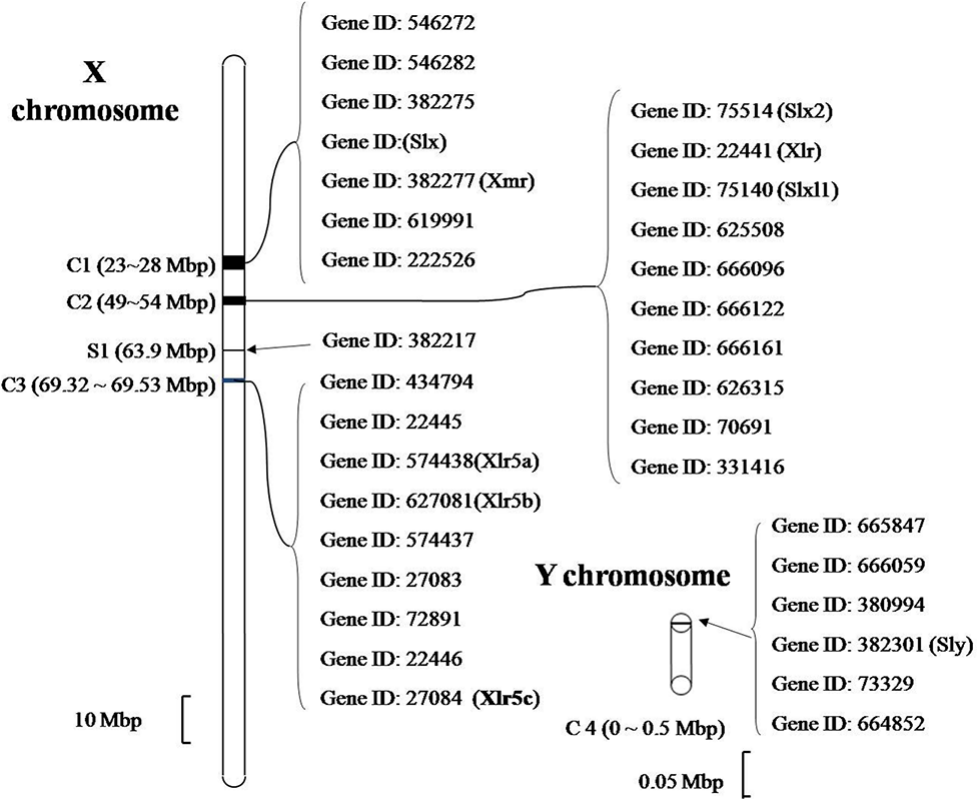
Representative members of the Xlr family mapped on the murine X and Y chromosomes. A total of 27 X-linked genes are divided into three clusters on the X and Y chromosomes, as determined using the PANTHER classification system. Cluster one contains seven genes including the well-studied Xmr and Slx, cluster two contains 10 genes including Slx2 and Xlr, and cluster 3 includes 9 genes from the Xlr4a-Xlr5c cluster. Cluster 4 contains Sly and five other members on the Y chromosome. The largest cluster on the X chromosome is approximately 3.5 Mbps, and the six Y chromosome genes form a small cluster of 0.4 Mbps.

According to the PANTHER classification system, Xlr5c belongs to the Xlr family which includes 34 genes identified from transcript profiling[4]. All Xlr family genes are on the X or the Y sex chromosomes except Sycp3, which is located on chromosome 10. The 27 X-linked genes formed three clusters and one solitary gene using the PANTHER classification system and DNAman software (Fig 1). Cluster 1 contains seven genes including the well-studied Xmr and Slx, cluster 2 contains 10 genes including Slx2 and Xlr, and cluster 3 includes 9 genes from the Xlr4a-Xlr5c cluster. Cluster 4 contains Sly and five other members on the Y chromosome. The largest cluster on the X chromosome is approximately 3.5 Mbps, and the six Y chromosome genes form a small cluster of 0.4 Mbps.

**Fig 2:**
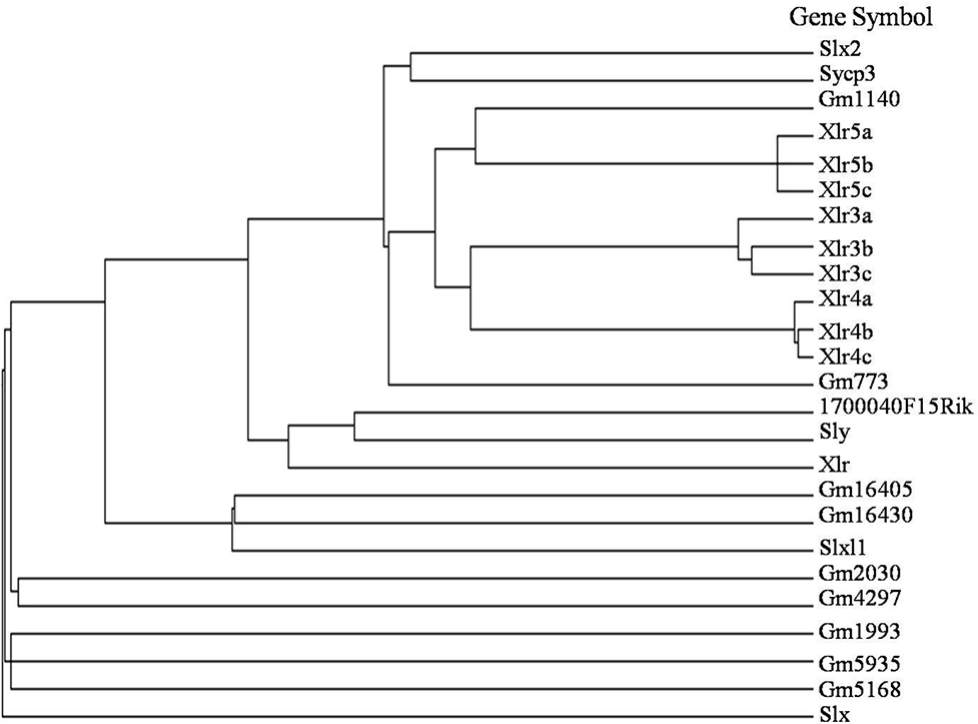
Multiple sequence alignment of representative members of the Xlr family. Phylogenetic tree of mouse Xlr family, was built using the MacVector molecular analysis program. Xlr5c is relatively distantly related to other members of the Xlr family, which contain a high proportion of basic residues and demonstrate significant homology to other members of the Xlr family.

**Fig 3:**
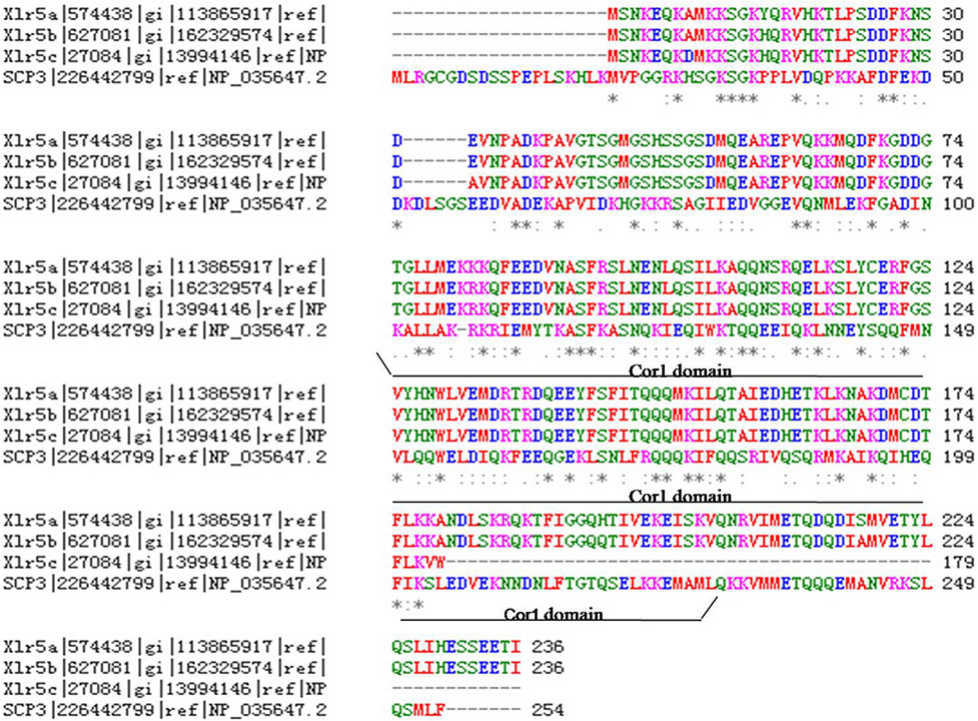
Amino acid sequence alignment of the Xlr5 subfamily and Sycp3. Xlr5c belongs to the Xlr5 subfamily, along with Xlr5a and Xlr5b according multiple sequence alignment. All three proteins share homology with Sycp3 and all include the conserved Cor1 domain.

Genome sequence data analysis shows that Xlr5c resides on the murine X chromosome at a locus entirely composed of regions with high sequence similarity to members of the Xlr gene families. Clusters in the mouse X chromosome region A7.1–A7.2 contain multiple duplications of an Xlr3-Xlr4-Xlr5 triad [2,4]. A FASTA comparison showed that Xlr5c is relatively distantly related to other members of the Xlr family, which contain a high proportion of basic residues and demonstrate significant homology to other members of the Xlr family (Fig 2). Xlr5c belongs to the Xlr5 subfamily, along with Xlr5a and Xlr5b. These proteins share a Cor1 domain similar to that of Sycp3 (Fig 3). Xlr5a consists of 236 residues and is highly homologous to Xlr5b (which also contains 236 residues; Fig 3). The Xlr5c cDNA is predicted to encode an ORF of 179 amino acids, which is shorter than Xlr5a and Xlr5b. However, all three proteins share homology with Sycp3, which is essential for the formation and maintenance of the lateral elements of the SC. These results indicated that they may perform a similar function in mouse tissues.

### *Xlr5c* transcripts are abundant in mouse testes

**Fig 4:**
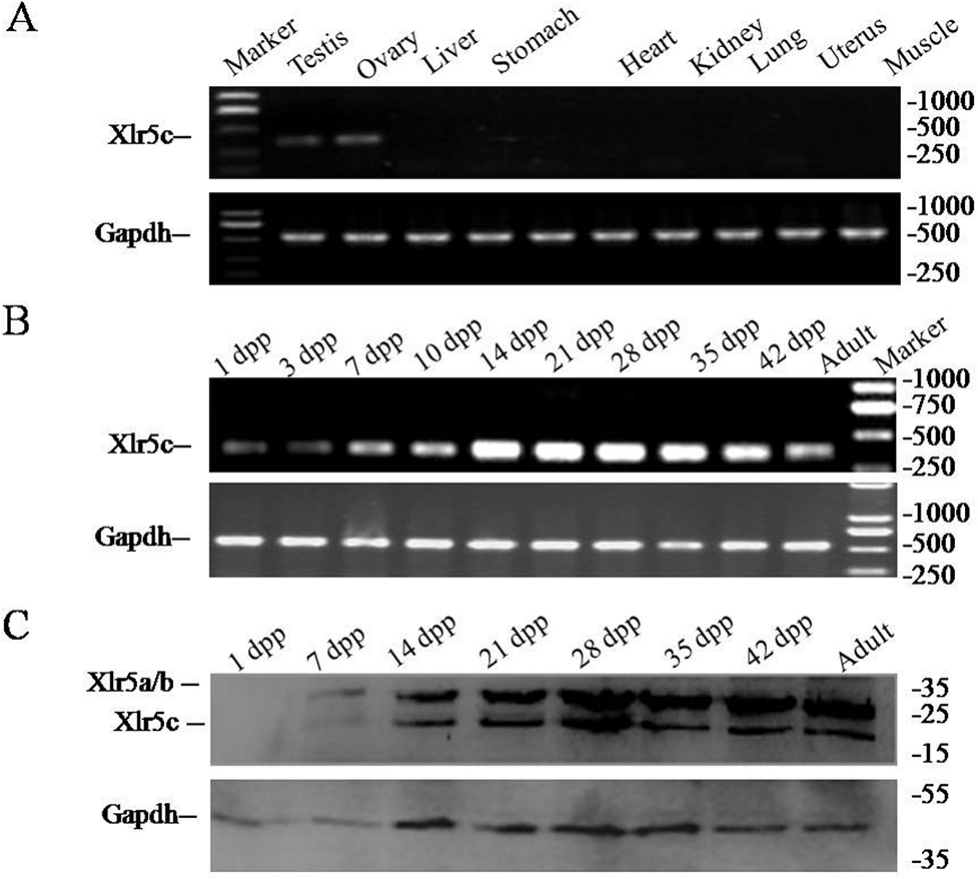
Transcription and translation of Xlr5c in mouse tissues and developing testes. (A) Position of primers used in RT-PCR experiments. Xlr5c mRNA is expressed exclusively in mouse testes and ovaries. (B) The Xlr5c transcript was first detected at 1 day post partum in postnatal mouse testes using RT-PCR. Expression levels increased gradually thereafter. (C) Two proteins of ~20 kDa and ~26 kDa were detected in testes using western blotting. The larger proteins correspond with Xlr5a and/or Xlr5b, and the smaller proteins correspond with Xlr5c. Gapdh mRNA and protein was used as an internal control.

Expression of Xlr5c was measured by RT-PCR using two pairs of primers (Table 1). Xlr5c mRNA was detected in the testes and ovaries (Fig 4A). Upon further investigation, Xlr5c expression was first detected one day post partum (dpp), and expression in testes increased gradually between 1 and 28 dpp (Fig 4B). Xlr5c was noticeable at 7 dpp, and expression continued to increase up to 14 dpp. These data indicated that Xlr5c was abundantly expressed in the developing testes.

Xlr5c antigen plasmids were designed and expressed in *E*. *coli* to generate polyclonal antibodies. Two polyclonal antibodies were prepared by immunizing rabbits with Xlr5c protein which were purified by protein-G affinity. Western blotting showed that two specific forms of protein were present in testes with a molecular size of ~20 kDa and ~26 kDa, respectively (Fig 4C). While the predicted mass of Xlr5c is only 20 kDa, we tried to identify the two forms by mass spectrometry. It turned out that the 20 kDa form was indeed Xlr5c, the 26 kDa was Xlr5a and/or Xlr5b.

### Xlr5c is a novel spermatogenesis-associated protein

**Fig 5:**
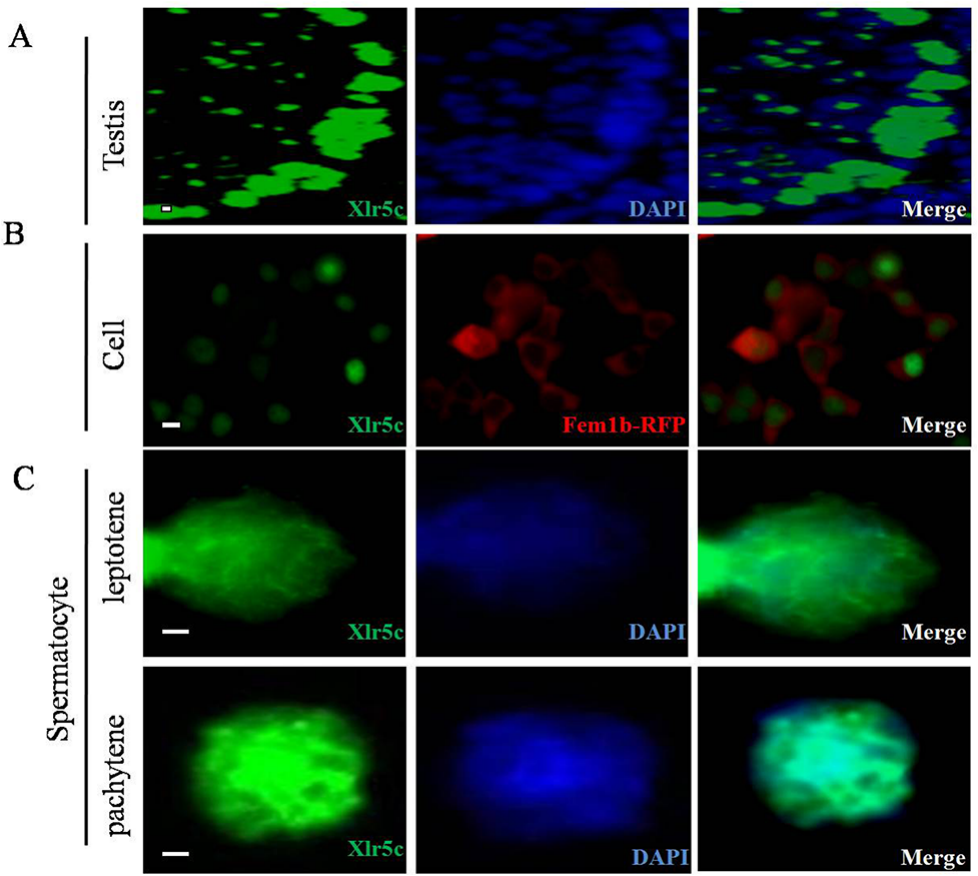
Localization of Xlr5c in testes and ovaries. (A) Co-staining of adult testes sections for Xlr5c (green) and DAPI (blue). Xlr5c was present in spermatocyte nuclei. (B) Subcellular localization of Xlr5c-GFP and thefusion Fem1b-RFP protein in CHO cells. Xlr5c-GFP was localized in the nuclei. (C) Xlr5c was most abundant during meiosis in spermatocytes. At the pachytene stage, Xlr5c was localized at the synaptonemal complexes, as shown previously for the homologous Sycp3. The red scale bar = 100 μm and the white scale bar = 10 μm.

To further examine the expression of Xlr5c in different germ cells, immunostaining was performed on gonadal sections, and expression was detected in mouse adult testicular sections (Fig 5A). Xlr5c was localized in dense regions that appeared as small bright dots in the nuclei of male germ cells, particularly in those undergoing spermatogenesis. This distribution was more apparent in some seminiferous tubules from the adult sections (Fig 5A). To further investigate the subcellular localization of Xlr5c, CHO cells were transfected with a plasmid expressing Xlr5c-EGFP and Fem1b-RFP. Fem1b is one of the mammalian homologs of the nematode C.elegans sex-determining gene Fem-1, whose fusion protein localized in the cytoplasmic of transfected cells. The Xlr5c fusion protein was located exclusively in the nuclei of transfected CHO cells (Fig 5B). The validity of the anti-Xlr5c antibody was proved as this successfully detected the Xlr5c-EGFP fusion proteins (data not shown). These results indicated that Xlr5c was a nuclear-localized protein may play an important role in spermatogenesis.

### Xlr5c may play a role in spermatogenesis

**Fig 6:**
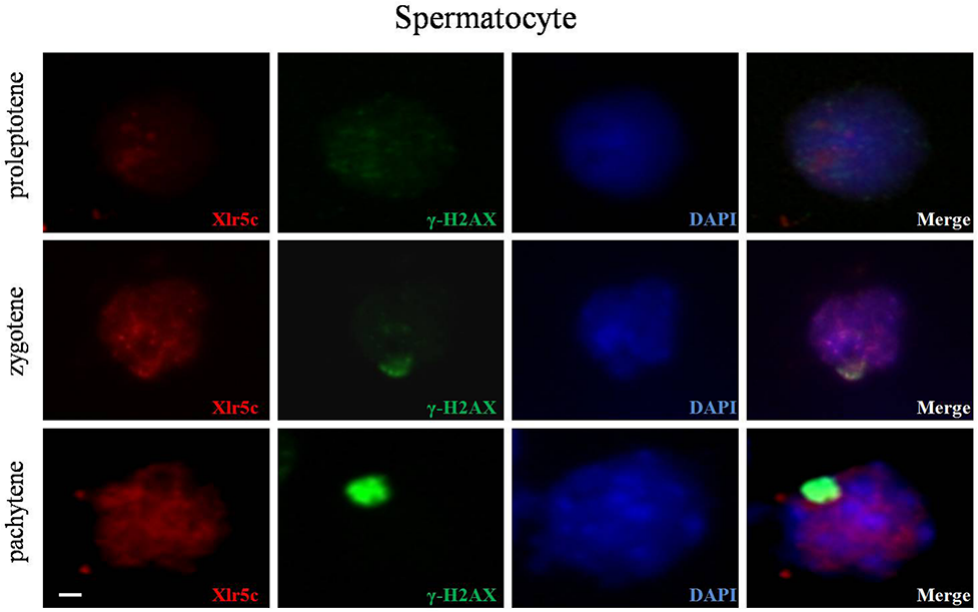
Co-localization of Xlr5c and γ-H2AX in chromosome sections. Xlr5c was localized in the nuclei of spermatocytes during meiosis. Xlr5c (red) and γ-H2AX (green) were co-localized in the chromosome sections of spermatocytes. Nuclei are stained with DAPI (blue). Scale bar = 10 μm.

Xlr5c mRNA was detected in testes by RT-PCR (Fig 4B), and expression levels increased significantly between 7 and 14 dpp, as spermatogenesis progresses into the meiotic stage. This suggested that Xlr5c may be involved in meiosis, therefore expression in adult testes at different stages of meiosis was investigated using immunostaining. Small strongly stained regions (spots) were observed in seminiferous tubules of the adult mice (Fig 5A). To further examine the expression of Xlr5c in different spermatocyte cells, immunostaining was performed on a preparation of chromosome spreads. Xlr5c was distributed uniformly across the nuclei of zygotene spermatocytes (Fig 5C), and was localized at the SCs region, as was the related Sycp3 in a previous study[6]. γH2AX is a marker protein which localization in the sex body during meiosis. Next, we probed the possible co-localization of Xlr5c and γ-H2AX, a well-known marker of the sex body in the chromosome spreads of spermatocytes. In preleptotene spermatocytes, the localization of Xlr5c in the spermatocyte nuclei closely matched the staining pattern of γ-H2AX (Fig 6). In pachytene spermatocytes, staining of γ-H2AX was restricted to the sex-body, however, Xlr5c was also localized to the SCs region. These results indicated that Xlr5c may participate in the meiosis stage of spermatogenesis.

## Discussion

The murine Xlr family was originally identified by subtractive cDNA hybridization [4] and found to play an important role in spermatogenesis. The Xlr superfamily has an estimated 50 ~75 copies per haploid genome, and functions in meiosis, post-meiotic maturation and fertilization of germ cells have been reported for various members, including Sycp3 [6], Slx2 [7], Xmr [10], Slxl1[12] and Sly [13]. In this study, Xlr5c was found to include the COR1 domain, and was localized in the nuclei of spermatocytes. To further investigate the subcellular localization of Xlr5c, CHO cells were transfected with a plasmid expressing Xlr5c-EGFP, and the fusion protein was exclusively located in the nuclei of transfected CHO cells. This suggested a nuclear localization, but further studies are needed to clarify whether expression occurs predominantly during prophase of the first meiotic division in male germ cells.

Sycp3 includes the conserved Cor1 domain and is essential for the formation and maintenance of the lateral elements of the SCs [6]. Xlr5c includes a conserved Cor1 domain and shares more similarity with Sycp3, suggesting a similar involvement in may be a putative meiosis protein-related protein. We hypothesized that Xlr5c acting as a novel meiosis-associate protein should play potential roles in regulation of meiotic processes Xlr5c was abundantly expressed in the testes, and both mRNA and protein were detected exclusively in meiotic spermatocytes. Xlr5c expression was first detected at 1dpp and increased by 7dpp. Xlr5c expression increased gradually up to 14 dpp, when cells in the first cycle of spermatogenesis are in the meiotic prophase, suggesting that Xlr5c may be involved in meiosis. Expression of Xlr5c was increased significantly by 14 dpp, when spermatogenesis had progressed to the pachytene stage, and was greatly increased at 21 dpp and thereafter, when spermatids are produced in the first wave of spermatogenesis. Although Xlr5c was localized within nuclei generally, its specific localization in the zygotene nuclei of spermatocytes suggested a possible involvement in meiosis in both sexes. Indeed, expression was also detected in the oocytes of females arrested at prophase during the first meiotic division. RT-PCR revealed that Xlr5c was expressed in the follicle of ovary sections.

Western blotting results revealed two protein forms (~20 kDa and ~26 kDa, respectively) in the testis recognized by the rabbit polyclonal antibody against the Xlr5c fragment. This was surprising given that the predicted molecular weight of Xlr5c was only ~20 kDa. The smaller of these proteins corresponds to the expected size of the 179 amino acid suggesting that Xlr5c protein migrates aberrantly in SDS PAGE, possibly due to modifications. Alternatively, the larger band may correspond with the expected size of Xlr5a and/or Xlr5b, which both contain 236 residues (~26 kDa). To further characterize the two bands, several modification experiments were performed. However, the presence of the 26kDa band was not affected by kinase treatment or preparation of extracts in the absence of protease inhibitors (data not shown), suggesting that modification were not due to phosphorylation, ubiqitination or sumoylation. Interestingly, Xlr5c shares higher homology with Xlr5a and Xlr5b than with other Xlr protein, and the particularly high similarity in the Cor1 domain may be explain the cross-reactivity of the anti-Xlr5c antibody with Xlr5a and Xlr5b. To further detect the western blot results, we tried to identify the two forms by mass spectrometry. It turned out that the 20 kDa form was indeed Xlr5c, the 26 kDa was Xlr5a and/or Xlr5b. These results explained that Xlr5a and/or Xlr5b were detected by the cross-reactivity of the anti- Xlr5c antibody. And the Xlr5c as well as Xlr5a and/or Xlr5b were co-localized in the nuclear of germ cells using the Xlr5c antibody and immunostaining.

Closer inspection showed that Xlr5c was localized at the SCs region in preleptotene and pachytene spermatocytes. This distribution is reminiscent of Sycp3, which is essential for the lateral elements of the SCs, and Xlr5c as well as Xlr5a and/or Xlr5b may well be associate with spermatogenesis.

In conclusion, murine Xlr5c was cloned, and its expression and localization in germ cells were investigated. The abundant expression in germ cells and the localization suggest that Xlr5c was a novel spermatogenesis-associated protein that may play an important role in this process.
